# The Motion Picture: Leveraging Movement to Enhance AI Object Detection in Ecology

**DOI:** 10.1002/ece3.71996

**Published:** 2025-08-19

**Authors:** Ben Maslen, Gordana Popovic, Dadong Wang, Andrew Jansen, David Warton

**Affiliations:** ^1^ School of Mathematics and Statistics University of New South Wales Sydney New South Wales Australia; ^2^ Evolution and Ecology Research Centre University of New South Wales Sydney New South Wales Australia; ^3^ Data61, CSIRO Eveleigh New South Wales Australia; ^4^ Mark Wainwright Analytical Centre University of New South Wales Sydney New South Wales Australia; ^5^ Department of Climate Change, Energy, Environment and Water Environmental Research Institute of the Supervising Scientist Darwin Northwest Territories Australia

**Keywords:** artificial intelligence, camera traps, computer vision, deep learning, ecology, machine learning, movement, underwater video

## Abstract

The rise of AI has seen an explosion in the use of deep learning methods that automate the analysis of image and video data, saving ecologists vast amounts of time and resources. Ecological imagery poses unique challenges; however, with cryptic species struggling to be detected among poor visibility and diverse environments. We propose leveraging movement information to attempt to improve the predictions produced by a high‐performing object detection algorithm. Frame differencing, background subtraction, optical flow and multi‐object tracking are trialed on four diverse datasets containing over 35,000 annotated images sourced from terrestrial, marine and freshwater habitats. We find that leveraging movement information is useful for smaller sized studies and rarer species, however is not needed for well annotated studies (> 400 annotations per class). Out of the methods that utilise movement, we find that a simple ‘differencing’ of neighbouring frames generally performed the best, whilst attempting to track taxa to boost prediction scores performed poorly. Other studies in this area tend to focus only on 1–2 datasets and a single method that utilises movement information, making it difficult for ecologists to generalise results. Our study provides key lessons for ecologists to determine whether it is useful to incorporate methods that leverage movement information when attempting to automatically predict taxa. We offer straightforward code for practical implementation via our GitHub repository, BenMaslen/MCD, along with an evaluation benchmark dataset called ‘Tassie BRUV’ that can be accessed from the Dryad public repository https://doi.org/10.5061/dryad.sbcc2frf7.

## Graphical abstract

As AI methods become increasingly popular to automatically analyse ecology data, we investigate using movement information as a means to improve traditional object detection approaches (which are limited to static image information only) on over 35,000 annotated images from 1480 terrestrial, marine and freshwater locations, the most extensive investigation to date. We find that leveraging movement patterns becomes more useful for smaller sized studies, however is not needed for well annotated studies (> 400 annotations per class). Our study provides key lessons for ecologists, as well as providing straightforward code for practical implementation via our GitHub repository BenMaslen/MCD and an evaluation benchmark dataset called ‘Tassie BRUV’.

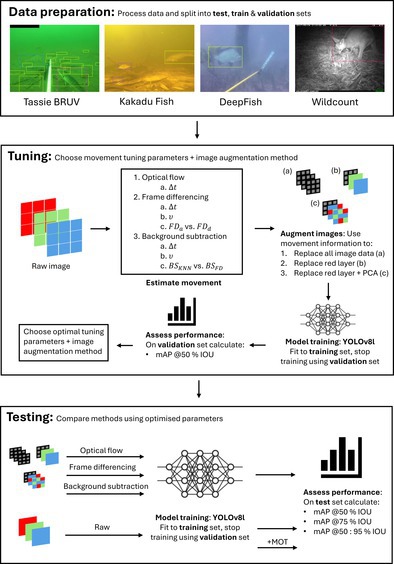

## 1 Introduction

In order to effectively manage our environment and assess the impact of ecological disturbances, it is imperative for ecologists to monitor and estimate the abundance of existing taxa. Modern technology has transformed the way ecologists collect these data in the field, via the use of remote monitoring tools like camera traps and monitoring videos (e.g., Walsh et al. 2016; Folpp et al. 2020; Steenweg et al. 2017; Oberosler et al. 2017). These methods are non‐invasive, relatively cheap to deploy and provide a permanent record of the recorded species and habitat (O'Connell et al. 2011; Rowcliffe et al. 2008; Johansson et al. 2008; Harvey et al. 2013). In the case of marine environments, it also allows ecologists to monitor habitats that were previously inaccessible due to harsh environmental factors like depth, swell, temperature, etc. (Johansson et al. 2008; Harvey et al. 2013). However, recording large amounts of photo and video data produces a new problem: how to analyse it all? Currently, the process is largely manual, where ecologists spend hours in front of a computer manually counting observed taxa (O'Connell et al. 2011; Johansson et al. 2008; Harvey et al. 2013). This process is incredibly time‐consuming and limits the scope of these monitoring tools for larger scale projects. Since the rise of deep learning algorithms, researchers have started to automate this process. However, this is typically done using still images, even when the original data collected were videos or burst shots (e.g., Siddiqui et al. 2018; Iqbal et al. 2021; Ditria et al. 2020; Connolly et al. 2021). This is because standard computer vision algorithms like YOLO or Mask R‐CNN take images as input (He et al. 2017; Redmon et al. 2016). Monitoring and classifying taxa in complex marine and terrestrial environments pose unique challenges; however, with cryptic taxa struggling to be detected accurately due to a wide range of environmental factors. A key factor for distinguishing cryptic animals from their surroundings is their movement patterns, so there is an opportunity to use movement information to improve performance.

A wide range of methods have been trialled historically that utilise movement to aid in the detection of taxa with object detection algorithms; however, there is little detail on which methods are particularly useful for ecological data. For these methods to be practically useful for ecologists, it is imperative that they satisfy three key criteria: (1) the method improves detection performance; (2) the method is easily implemented by ecologists; and (3) the method can adapt to future technological changes. For criterion (1), we define detection performance as having minimal false positives, false negatives and misclassifications when predicting taxa. An effective metric (mean Average Precision—mAP) of performance is discussed later in the results section. With object detection algorithms and artificial intelligence improving rapidly (Zou et al. 2023), criterion (3) ensures that ecologists are able to apply these methods to the best detection model at the time. To satisfy criterion (3), it may be advantageous to incorporate movement information by augmenting the image data to better fit existing models (e.g., Bloice et al. 2019) or to incorporate movement by post‐processing model output (e.g., through tracking algorithms, Zhou et al. 2020; Zhang et al. 2021, 2022). The alternative, altering currently existing model architectures to handle additional input layers of movement information, has the problem that it is not ‘future‐proofed’—our work would be specific to a given model architecture, so the process would need to be restarted any time an improved architecture was developed that we wanted to use. Post‐processing techniques such as the Hungarian algorithm, Greedy algorithm, Kalman filtering or tracking algorithms (which use movement information indirectly) have been used to link detections between frames (Li et al. 2020; Panadeiro et al. 2021; Lopez‐Marcano et al. 2021; Wageeh et al. 2021; Mohamed et al. 2020). Some researchers have taken this a step further by using tracking or temporal movement information to categorise the behaviour of taxa in monitoring videos (Måløy et al. 2019; Ditria et al. 2021; McIntosh et al. 2020; Huang et al. 2022; Lopez‐Marcano et al. 2021). Another way video movement information has been utilised in ecology is to detect when an animal is on screen, to either thin down data for manual labelling or to trigger an object detection algorithm on the frames that have animals (Coro and Bjerregaard Walsh 2021; Golkarnarenji et al. 2018; Nazir et al. 2019; Weinstein 2015). This becomes particularly important for motion detection camera trap data to filter large amounts of misfired images caused by environmental factors (e.g., wind, cloud, shadows etc.), with the MegaDetector algorithm being a popular method for doing this (Weiss et al. 2016). Outside of the world of ecology, there are more advanced temporal detection algorithms that incorporate historic, future frame information or both into the architecture of the object detector (e.g., Deng et al. 2019; Beery et al. 2020; Shvets et al. 2019) or that track and identify objects jointly throughout a video using 3D architectures (e.g., ‘tubelet detectors’—Kalogeiton et al. 2017; Kang et al. 2016; Köpüklü et al. 2020). These methods, however, require large amounts of training data, trained in ‘tubelets’ (sequences of frames) for each species or class. This poses a problem for ecologists, who often collect data containing a large number of species, many of which are present in low quantities. Considering the additional investment in annotating more training data, this approach may not be practical for broad‐scale application. Movement can also be measured directly and used as a predictor or feature in an object detection algorithm by either adding extra layers to an image or a frame (made up of three colour layers RGB—red, green and blue) and altering the architecture of the detection algorithm to handle more than three layers of input data per image or by augmenting the image/frame data to include movement information. The latter requires no structural changes to the model architecture and so can use ‘off‐the‐shelf’ any modern algorithm developed for object detection from images.

In this study, we focus on methods that ecologists can easily implement and adapt to future technological changes, whilst assessing their improvement to detection accuracy, thus satisfying our three aforementioned requirements. We trial a combination of different measures of movement to augment training data to feed into the high performing YOLOv8 object detection algorithm (Jocher et al. 2023). This will be compared to feeding in the raw image data alone, as well as linking the object detections using a tracking algorithm. These methods will be trialled on four distinct underwater video monitoring and terrestrial camera trap studies to assess their practical use by ecologists. These methods were chosen as they do not require additional training data to be collected, beyond that already needed to train a classifier that uses raw images, and the methods are relatively simple for ecologists to use in practice. Researchers have attempted to use these methods in a variety of ways in underwater monitoring and camera trap studies (Salman et al. 2020; Jalal et al. 2020; Seese et al. 2016; Schindler and Steinhage 2021; Zhang et al. 2016; Duhayyim et al. 2021); what is lacking, however, from current literature is the direct comparison of these methods across a range of differing ecological monitoring datasets (with most studies only trialling 1–2 methods on 1–2 datasets). This will better inform ecologists on whether these methods are worthwhile and which one is the most effective to utilise in practice. Movement can be estimated in a variety of ways: by directly estimating the velocity of pixels across neighbouring frames (optical flow); by observing the difference in pixel values across neighbouring frames (frame differencing); or by separating pixels into foreground and background groups (background subtraction). We will be trialling all of these methods as well as a combination of ways to augment images through replacing uninformative colour layers and dimension reduction through principal component analysis. We will also be using the ByteTrack tracking algorithm to link detections across frames as a trial of a movement‐oriented post processing technique. Through this comparison, we will be able to guide ecologists on whether utilising movement information is worthwhile for their data and which approach works best.

## 2 Materials and Methods

### 2.1 Data

**FIGURE 1:**
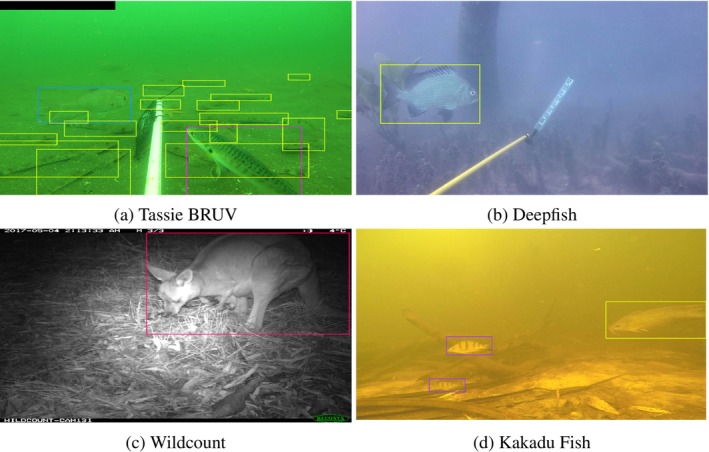
Annotated frames from the Tassie BRUV (a), Deepfish (b), Wildcount (c) and Kakadu Fish (d) training sets. Note the baited mesh bag in the Tassie BRUV monitoring video and the monochrome night shot in the Wildcount image (majority of Wildcount images were taken at night from nocturnal animals).

Four datasets were used which link videos and burst shots to 38,287 annotated images. These images were sourced from over 450 h of video footage and 1.5 million burst images from 1480 diverse marine, freshwater and terrestrial locations across Australia. Images were labelled with a total of 52,284 annotations classified into 36 classes. Example images are shown in Figure 1, with a summary of key dataset properties found in Table 1 and data processing in the Data preparation section of the S1.

**TABLE 1:** Metadata for the datasets used in this study.

Data	Surrounding videos/images	Type	Sites	Species	Classes	Annotations	Labelled images
Tassie BRUV	28	UMV	28	19	7	5222	1912
Kakadu Fish	352	UMV	39	19	4	19,957	8960
Deepfish	39,766	UMV	19	> 1	1	310	620
Wildcount	1,488,820	CT	1394	24	24	26,795	26,795

*Note:* Sites include each locality where Underwater Monitoring Videos (UMV) and Camera Traps (CT) were placed. The Deepfish (Saleh et al. 2020) and Wildcount (McSorley et al. 2023; McHugh et al. 2022; NPWS 2025) datasets have surrounding images, whilst the Tassie BRUV and Kakadu Fish (Jansen et al. 2024; SSD 2011) datasets have surrounding videos. For more details, refer to Figure 1 and the Data preparation section in the Supporting Information.

Although only using four datasets, this is still the most comprehensive set of benchmark datasets assembled to date that investigates the effectiveness of using movement information to aid in the detection of taxa in ecological monitoring videos and images. Assembling these datasets is difficult as there is a distinct lack of publicly available data that have been reliably labelled with access to both their surrounding videos/burst images as well as the location of the labelled images within these surrounding videos/images (which is imperative to obtain movement information). As such, almost all studies investigating the effectiveness of using movement information to aid in the detection of taxa have used only 1–2 datasets and focus on either underwater monitoring videos or camera trap studies separately (e.g., Li et al. 2020; Lopez‐Marcano et al. 2021; Salman et al. 2020; Jalal et al. 2020; Seese et al. 2016; Schindler and Steinhage 2021; Zhang et al. 2016; Duhayyim et al. 2021). To aid in this area, we have also manually annotated our own dataset (Tassie BRUV), which comprises 5222 annotations on 1912 frames sourced from 28 Baited Remote Underwater Videos (BRUVs) in Tasmania. This dataset can be used as a benchmark to develop object detection and movement quantification methods for fish in underwater videos. It can be accessed from the Dryad public repository (https://doi.org/10.5061/dryad.sbcc2frf7).

Annotated images from each of the datasets were split into training, test and validation sets (see Data preparation section in the Supporting Information for further details) to trial a range of methods to measure movement (optical flow, frame differencing and background subtraction). Movement augmented images were then used to train a deep learning model with the YOLOv8l detection algorithm (Jocher et al. 2023) in addition to the original ‘raw’ images. This was done by: first creating movement augmented copies of the labelled datasets; then (for each augmented copy as well as the original images) YOLOv8l model weights were estimated by fitting the model to the ‘train’ split, stopping training when loss is minimised on the ‘validation’ split; then the ‘test’ split was used to assess predictive performance (see Figure 4). Pre‐trained model weights were used as a starting point for model training to take advantage of ‘transfer learning’ (Weiss et al. 2016). Although the YOLOv8l model is pre‐trained on a large image dataset, by re‐estimating the model weights using augmented movement images, we are training the model to predict changes in movement. Previous studies have also used this approach 1080×1080thermal images (e.g., Ivasić‐Kos et al. 2019). The images were resized to pixels before being fed into the model and the default setting parameters were used when training (e.g., batch size of 16, initial learning rate of 0.01 etc., Jocher et al. 2023). The number of epochs required for loss to be minimised in the ‘validation split’ differed depending on the dataset and augmentation method, although generally ranged from around 100–300 epochs. The detections from the raw images were then altered using movement indirectly through the Bytetrack tracking algorithm (Zhang et al. 2022). Finally the prediction results were compared across all of these methods using the test splits from each dataset. There are many tuning parameters and choices that can be specified when utilising these movement approaches and deciding on how to augment the images. It is not realistic to exhaustively trial all the methods and tuning parameters for each movement quantification approach, instead we chose a set of methods and tuning parameters that would be relatively quick and easy for ecologists to use in practice, and that have been shown to work effectively in literature. These tuning parameters and methods were then optimised using the validation sets from each dataset. Below we will detail a short description of the different movement quantification methods utilised with visualisations of how they augment the images in Figure 1, as well as the tuning, tracking and testing approaches we implemented.

### 2.2 Optical Flow

Optical flow is the velocity $\vec{v}$ of pixels between frames in a video (or images in a burst camera shot) of distance Δt in time. Optical flow methods attempt to estimate the direction θ and magnitude r of pixel motion, where motion can be caused by objects moving within a frame or the camera itself moving, by measuring the change in brightness at time t, for pixel locations $\left(x,y\right)$ in frame $F_{t}$.

$$\begin{array}{l} \vec{v}=\left(\begin{array}{c} v_{x}\\ v_{y} \end{array}\right)=\left(\begin{array}{c} \frac{\Delta x}{\Delta t}\\ \frac{\Delta y}{\Delta t} \end{array}\right)\\ \Rightarrow r=\sqrt{v_{x}^{2}+v_{y}^{2}}\;\&\;\theta =\tan^{-1}\left(\frac{v_{x}}{v_{y}}\right) \end{array}$$

Estimating optical flow directly is not plausible; however, due to the undetermined aperture problem where the change in brightness alone can not uniquely determine both the direction θ and magnitude r of pixel flow (Shah and Xuezhi 2021). Current methods to overcome this and estimate optical flow can be split into two main categories: traditional methods that introduce additional constraints and rely on theory based assumptions regarding the smoothness and consistency of brightness for feature evaluation; and deep learning based approaches that use large amounts of optical flow training data to train a convolutional neural network or other deep learning model to estimate optical flow (Shah and Xuezhi 2021).

We estimated optical flow using the Farneback algorithm (Farnebäck 2003), due to its simple and effective implementation through the popular OpenCV library in Python (Bradski and Kaehler 2000) and success in other studies (Golkarnarenji et al. 2018). The Farneback algorithm assumes that neighbouring pixels have similar motion vectors and estimates these motion vectors using a polynomial expansion transform (Farnebäck 2003).

### 2.3 Frame Differencing

Frame differencing is the process of subtracting neighbouring frames in a video. If an object is moving in a video with a static background, the object's pixel values will change whilst the static background will not. Therefore by taking the difference between two frames of distance Δt apart, any change in a pixel's colour value is indicative of movement. Taking the absolute value of differences is generally chosen (Singla 2014) and this can be done using a single ‘neighbouring’ frame or using frames either side of a frame of interest as a ‘three frame difference’ (which we implemented). A frame of height h and width w is comprised of an h×w×3 array of Red, Green and Blue (RGB) colour values for each pixel in the frame. If we take a frame $F_{t}$ of pixels at time‐point t of interest, a three frame difference is calculated by averaging the absolute difference between frame $F_{t}$ and neighbouring frames $F_{t\pm \Delta t}$:

$${\mathrm{FD}}_{a}=\frac{1}{2}\left(|F_{t}-F_{t-\Delta t}|+|F_{t}-F_{t+\Delta t}|\right)$$

Researchers will then generally convert this into a binary result using a threshold value to decide what is and is not moving (Singla 2014). For our use in movement quantification for object detection, this process would be needlessly removing information that can describe the scale of motion. Leaving it as a raw value allows the object detection model to decide using our labelled training data what values of the frame difference is useful in deciding what is and is not moving. We also trialled increasing the signal of motion detection by multiplying the frame difference by a constant υ and truncating the resultant values to be within their normalised range of 0–1.

As an alternative to the absolute value based approach to frame differencing, we also measured the direction of motion, by averaging the difference of the left frame $F_{t-\Delta t}$ to the centre frame $F_{t}$ as well as the left frame to the right frame $F_{t+\Delta t}$ and centring it at 0.5:

$${\mathrm{FD}}_{d}^{\upsilon }=\frac{1}{2}+\frac{\upsilon }{4}\left(F_{t}-F_{t-\Delta t}+F_{t+\Delta t}-F_{t-\Delta t}\right)$$

This method allows any pixels that have a difference above 0.5 to be increasing in its associated colour value, and below 0.5 to be decreasing from frame $F_{t-\Delta t}$ to $F_{t}$ and $F_{t+\Delta t}$.

When implementing frame differencing, we thus have three parameters to tune:

1. υ, the amount by which difference signals are scaled. Values larger than one might assist detection.

2. Method of computation, either using an absolute value approach (${\mathrm{FD}}_{a}$) or a directional approach (${\mathrm{FD}}_{d}$).

3. Δt, the time difference between the neighbouring frames $F_{t\pm \Delta t}$ and frame of interest $F_{t}$. Larger values accentuate movement, providing a stronger signal but with less spatial precision.

### 2.4 Background Subtraction

Background subtraction is a technique used to extract foreground objects from a video sequence by differentiating them from a static background. Thus, it is indirectly evaluating what is moving in a video, by first working out what is not moving (i.e., the ‘background’) in a video and observing what pixels are different from this background model (i.e., the ‘foreground’) in a frame of interest. Assuming we have a static background, these foreground pixels can then be classed as pixels that are currently moving or have recently moved into a new position relative to the static background. Particularly before the rise of neural networks, background subtraction was seen as a favourable unsupervised learning technique for object detection and segmentation in video data (Piccardi 2004). It has also been used for a variety of other applications like object tracking, surveillance systems and activity recognition (Piccardi 2004). There are many existing algorithms for estimating background subtraction (Stauffer and Grimson 1999; Braham and Van Droogenbroeck 2016; Barnich and Van Droogenbroeck 2011; Peterson 2009). We used a K‐nearest neighbours based (KNN) approach (Peterson 2009), due to its simple implementation within the popular open‐CV library in Python (Bradski and Kaehler 2000) and success in other studies (Trnovszký et al. 2017; Yasir and Yossra 2022).

The KNN algorithm takes pixels from historic frames as reference samples for the background class. Then, for a pixel in a new frame, surrounding pixels or pixels recently classed as foreground pixels are extracted as samples for the foreground class. Next, the distance (Euclidean or otherwise) is calculated between the new pixel's colour values and those from the background and foreground reference samples, and the K nearest pixels (neighbours) are selected. Finally, whichever class the majority of pixels among the k‐nearest neighbours belong to is the class that is chosen for the new pixel (Figure 3).

Traditional background subtraction methods give us a binary outcome on whether a pixel is assumed to be background or foreground. We also trialled another approach, measuring what could be considered to be background/foreground on a scale and allowing the training data to guide a neural network‐based object detection model in deciding the appropriate cut‐offs for each class. We did this by taking a frame‐differencing‐based approach.

If we take a h×w×3 frame $F_{t}$ of pixels at time‐point t of interest, we obtained an approximate background frame $F_{b}$ by ‘averaging out’ the foreground objects across $n_{b}$ background frames, each with time $\Delta t_{b}$ apart:

$$F_{b}=\frac{1}{n_{b}}\sum_{i}^{n_{b}}F_{t-i\Delta t_{b}}$$

for integer values of i ranging from 1 to $n_{b}$ (although background frames do not necessarily need to be historic).

Then, similar to the directed frame differencing approach, we obtain a measure of movement BS_FD_ as the distance from this background frame by subtracting it from our frame of interest, centred at 0.5 (which we also trialled scaling by a scaling parameter υ):

$${\mathrm{BS}}_{\mathrm{FD}}=\frac{1}{2}+\frac{\upsilon }{2}\left(F_{t}-F_{b}\right)$$

This is a simple method for estimating a background subtraction predictor that is quick and simple to implement. Being on a continuous scale, we are also able to capitalise on the training data deciding what is moving or not.

### 2.5 Image Augmentation

**FIGURE 2:**
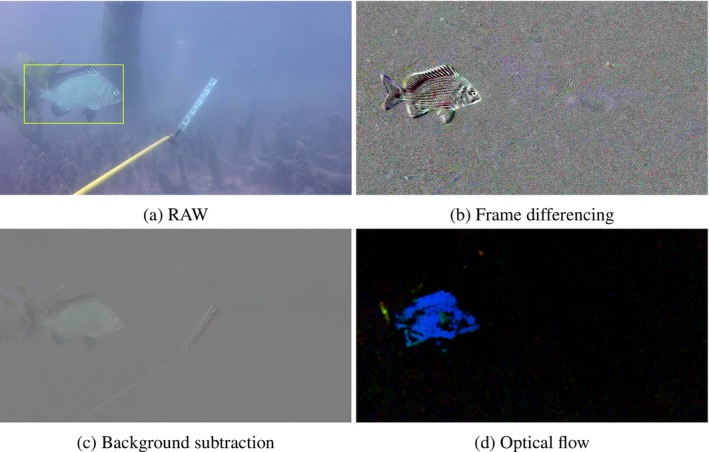
Examples of image movement quantification methods with original raw annotated image from the Deepfish dataset (a), frame differencing (b), background subtraction through frame differencing (c) and optical flow (d).

Now that we have introduced techniques to measure movement, we now need to use them as ‘predictors’ or ‘features’ in an object detection algorithm. However, standard algorithms such as YOLOv8 are designed to take three layers (the RGB colour layers that comprise an image or a frame) as input (Jocher et al. 2023). We could restructure an existing algorithm to handle more than three layers as input; however, this work would then need to be duplicated every time a new object detection algorithm is created. With new algorithms constantly being created with improved prediction accuracy (Zou et al. 2023), this would make any approach that uses movement unable to adapt easily to future technological changes. Instead, we can augment the RGB colour layers in an image to incorporate movement information. Augmenting image data is a common preprocessing technique for machine learning algorithms, widely used in biomedical imaging, for example, where large amounts of labelled data are difficult to come by or expensive to produce (Bloice et al. 2019). Using the aforementioned methods to measure movement, we trialled altering a frame of interest by replacing or augmenting some or even all of the RGB colour layers with measures of movement (as in Figure 2).

**FIGURE 3:**
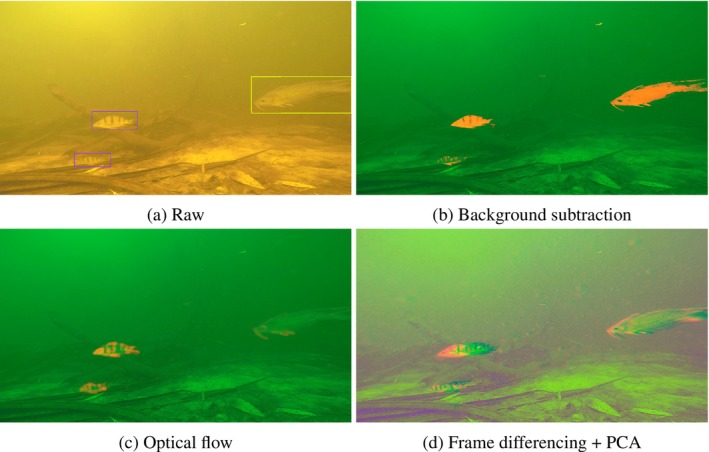
Augmented images on an annotated image from the Kakadu Fish dataset (a), after replacing the red colour layer with background subtraction (b), optical flow (c), and frame differencing (d). The green and blue layers of the bottom right image have also been replaced with a dimension reduced transform of the original three colour layers using PCA.

As light travels through water, the lower energy, longer wavelengths get absorbed by water molecules more easily then shorter wavelengths. For visible light, this removes a lot of the red colouration, leaving only blue and green light (up until a depth where even these colours are removed). Consequently, frames coming from underwater monitoring videos can be expected to have much smaller values in the red part of the RGB colour matrix. As such, we trialled removing these already small values by replacing the red layer with movement information. Thus, we augmented images to have two layers of colour information (the blue and green h×w layers) and another of movement information (e.g., Figure 3). To do this, we will need movement information as a single h×w matrix. This is done automatically for KNN background subtraction (Figure 3), so we have nothing to apply here. However, optical flow, frame differencing, and a frame differencing based approach to background subtraction produce a h×w×3 matrix of movement information by default. We reduced frame differencing and a frame differencing based approach to background subtraction to a single layer by averaging the three layers together. Optical flow on the other hand can be converted to a single layer by including only the magnitude r of flow, removing the direction information (Figure 3).

Another approach to diminish the loss of information caused by removing colour information is to use principal component analysis (PCA) as a dimension reduction technique to convert three layers of RGB colour information to two. This again frees up one layer for movement information, which we fill as previously. To ensure that the dimension reduction is consistent across all images in a dataset, we start by taking a random sample $n_{t}$ (or all) of images from the training set, rearranging them into single layer matrices of size hw×3 and append them all into a single long matrix of size ${\mathrm{hwn}}_{t}\times 3$ (thus generating a matrix where each row is a new pixel from $n_{t}$ images, and each column is a different colour). Then we undergo principal component analysis combined with whitening (to maintain intrinsic features of the image and reduce correlated noise—Shi et al. 2016; Hyvärinen et al. 2009) using the scikit‐learn library in Python (Pedregosa et al. 2011) to find the optimal principal components $p_{1},p_{2}$ that converts the three‐colour matrix into a two‐colour matrix of size ${\mathrm{hwn}}_{t}\times 2$. Finally, using the estimated principal components $p_{1},p_{2}$, we trialled converting our h×w×3 RGB colour images into dimension reduced colour images of size h×w×2, freeing up one layer in our images for movement information (Figure 3).

### 2.6 Tuning

**FIGURE 4:**
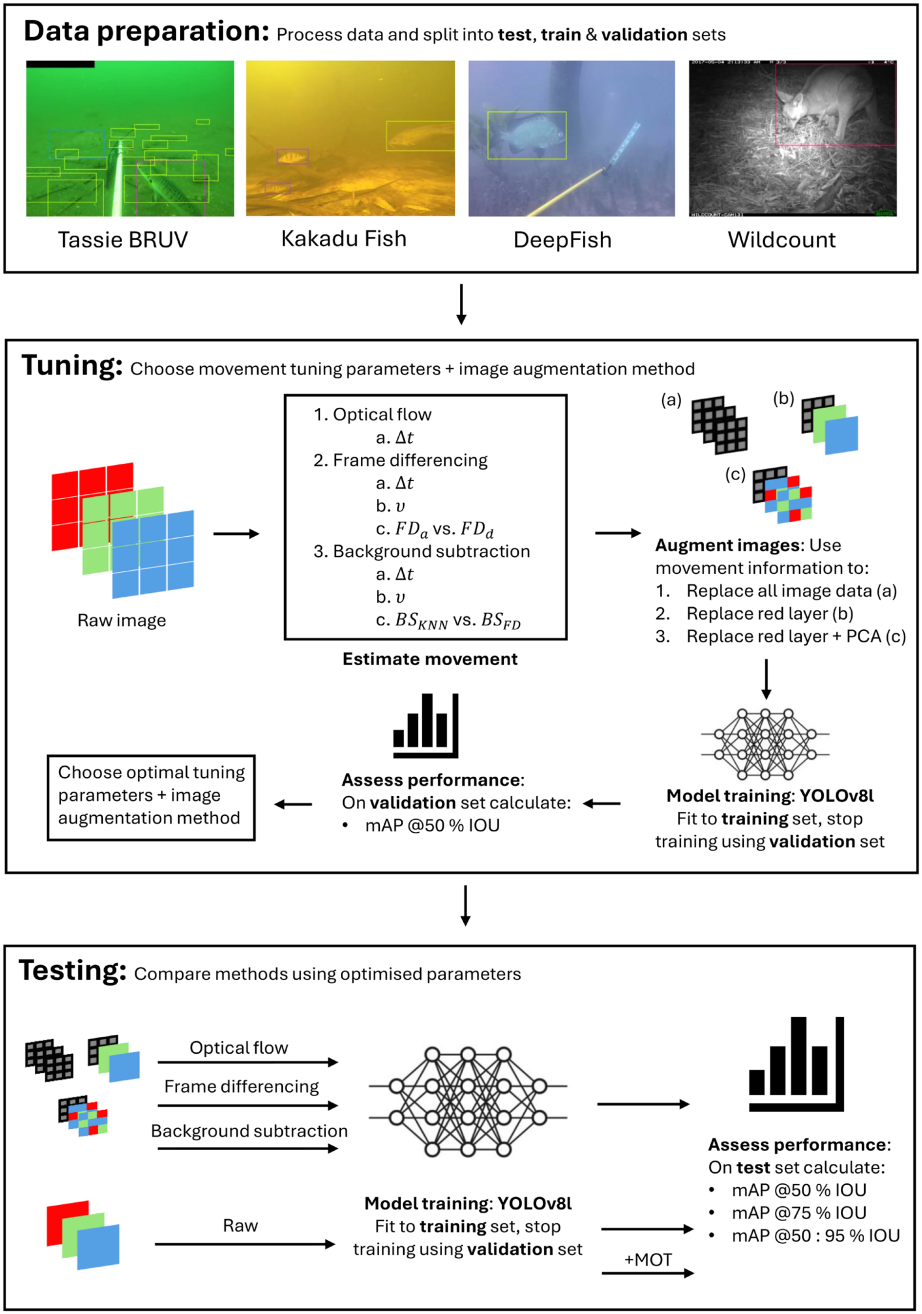
Methodological workflow for preparing data, tuning and testing. The tuning and testing procedures were undergone separately for each dataset. ‘+MOT’ refers to altering raw predictions with Multi‐Object Tracking. Refer to Table 2 for more details about tuning parameters (Δt, υ, FD_a_, FD_d_, BS_KNN_, BS_FD_).

The parameters for each movement detection, and the decision about which augmentation method to use, were tuned separately for each dataset (Figure 4). To avoid over fitting, this was done by observing the predictive performance on the validation split of the data, whilst the testing split was reserved for assessing the overall performance of each method post tuning. Movement detection parameters were tuned first, and then using these tuned parameters, an optimal image augmentation approach was chosen. Table 2 summarises all the chosen parameters and image augmentation approaches that were tuned for each movement detection approach. Frame differencing using a direction FD_d_ or absolute value FD_a_ based approach was compared as well as a range of values for Δt (number of frames between the differenced frame and the reference frame) and υ (differencing scaling parameter). Optical flow $\vec{v}$ with a range of values for Δt was compared, and background subtraction using KNN and a frame differencing based approach with the same υ chosen from frame differencing was compared with appropriate parameters for frame differencing chosen based on the number of background images in each dataset and the tuning of the frame differencing method. Using the tuned parameters for each movement detection approach, the image augmentation process was again tuned using the validation set. This was done by comparing the results on the validation set when either replacing all the image data with movement information, only the red layer with movement information or using PCA to dimension reduce the three colour layers to two and filling the third layer with movement information. This process produced an optimal tuning set for each movement detection and augmentation process for each dataset, with the results shown in Table 2 (see Figure 4 for a workflow diagram).

**TABLE 2:** Chosen tuning parameters for each movement estimation process, based on the optimal performance on the validation splits.

Data	Method	Δt	υ	Approach	Augmentation
Deepfish	Optical flow	1	NA	NA	Red
	Background subtraction	1	1	BS_FD_	All
	Frame differencing	1	1	FD_a_	All
Wildcount	Optical flow	1	NA	NA	Red
	Background subtraction	1	1	BS_FD_	PCA
	Frame differencing	1	1	FD_a_	Red
Kakadu Fish	Optical flow	5	NA	NA	PCA
	Background subtraction	100	15	BS_FD_	PCA
	Frame differencing	5	15	FD_d_	PCA
Tassie BRUV	Optical flow	1	NA	NA	PCA
	Background subtraction	120	15	BS_FD_	PCA
	Frame differencing	4	15	FD_d_	PCA

*Note:* Δt and υ is the time difference (in frames) between neighbouring frames and differencing scale parameter, respectively. BS_FD_ refers to background subtraction through frame differencing, FD_a_ and FD_d_ refers to frame differencing through absolute value and direction based approaches, whilst ‘all’, ‘red’ and ‘PCA’ refers to augmenting all the image data, only the red layer, and dimension reducing with PCA, respectively.

### 2.7 Tracking

Multi‐Object Tracking (MOT) algorithms aim to link detected objects across frames in a video to obtain a unique ID for each tracked object. This is traditionally done by first fitting a standard object detection algorithm to a video and obtaining predicted detections for each frame. Next, the tracking algorithm aims to link detections across frames by predicting the object's location at a current frame based on previous frame tracklets (referred to as tracking‐by‐detection). Recently, methods have also been developed that detect and track objects jointly in a unified framework (Zhang et al. 2021; Zhou et al. 2020), or that take advantage of transformer‐based models as a means to improve tracking accuracy (Zeng et al. 2022). On top of providing unique IDs to each object, tracking algorithms are thought to improve the overall detection accuracy by boosting scores of weaker detections that are neighbouring stronger detections (Li et al. 2020; Zhang et al. 2022). For example, if a fish were to change its pose to a point where it is difficult to ID on a certain frame, yet was easier to ID on neighbouring frames when its pose was perpendicular to the camera, a tracking algorithm could boost the lower confidence scores of the harder‐to‐ID pose. By adjusting detection scores across frames, this is indirectly using movement to aid in the detection of objects in a video.

We used the ByteTrack algorithm due to its high performance on MOT benchmark datasets MOT17 and MOT20 (Zhang et al. 2022), as well as its simple application within the YOLOv8 object detection framework (Jocher et al. 2023). Another benefit of ByteTrack is that it does not ignore low confidence detections when associating detections and developing tracklets using its developed ‘Byte’ algorithm (Zhang et al. 2022). This is something that other tracking algorithms ignore and can aid in the detection of schooling or occluded objects (Zhang et al. 2022), which is common in ecological monitoring videos. As MOT algorithms require videos to develop effective tracklets across frames, the ByteTrack algorithm will only be applied to the Kakadu Fish and Tassie BRUV datasets, which have surrounding videos.

### 2.8 Testing

To assess and compare the methods, predictive performance was evaluated on a holdout test split from each dataset (after each method had been tuned using the validation splits). Thus, we are able to compare predictions that incorporate frame differencing, background subtraction, optical flow and tracking, to just using the raw image data on its own (Figure 4). We utilised the accessible open‐source toolkit for object detection metrics produced by Padilla et al. (2021), to ensure that our results are replicable and comparable across other studies. Predictive performance was measured using mean Average Precision (mAP) at 50% Intersection Over Union (mAP @50% IOU), 75% Intersection Over Union (mAP @75% IOU), as well as average mAP from IOU = 50%. 55%, …, 95% (mAP @50:95% IOU). Intersection Over Union (IOU) refers to the percentage overlap of area between the predicted bounding box, and true bounding box, whilst mean Average Precision (mAP) refers to the average precision ($\frac{\text{True Positives}}{\text{True Positives}+\text{True Negatives}}$), calculated across all prediction thresholds. These metrics were chosen as they are considered industry standard, and are regularly used to benchmark methodology (Padilla et al. 2021).

## 3 Results

**FIGURE 5:**
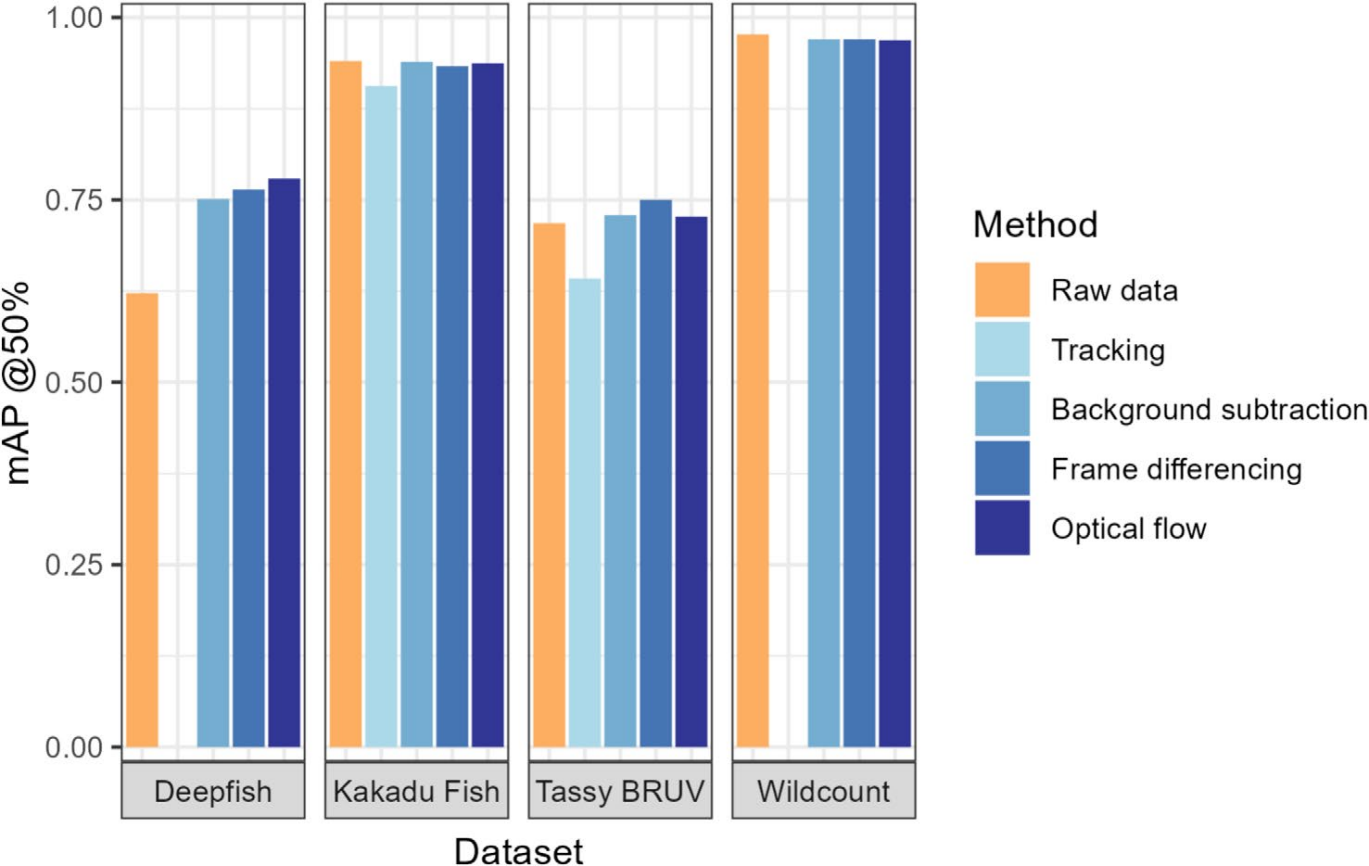
mAP @50% IOU results on the test set from the Deepfish, Kakadu Fish, Tassie BRUV and Wildcount datasets using a YOLOv8l object detection algorithm. ‘Raw’ refers to training using the original unmodified image data. Tracking was only able to be performed on the Tassie BRUV and Kakadu Fish datasets which had surrounding videos.

Results varied across the datasets. For the smaller datasets (Deepfish and Tassie BRUV), there was an increase in detection performance when using almost any movement augmentation approach (Table 3 and Figure 5), whilst for the larger datasets (Wildcount and Kakadu Fish), there was no increase in detection performance using movement information. Frame differencing, the simplest movement augmentation approach, managed to perform the best in terms of mAP @75% IOU and mAP @50:95% IOU for the Deepfish and Tassy BRUV datasets, and in terms of mAP @50% IOU for the Tassy BRUV dataset (Table 3 and Figure 5). Optical flow had the highest mAP @ 50% IOU score for the Deepfish dataset, whilst using raw umodified images performed the best across all metrics for the Kakadu Fish and Wildcount data sets (Table 3 and Figure 5). Improvement to detection accuracy using movement information seems to be directly related to the sample size of the dataset. The smallest dataset, Deepfish (n=620), had the largest increase in mAP @50% IOU (23%) when using optical flow rather than using only the raw image data. Conversely, the largest dataset, Wildcount (n=26,795) had a reduction in mAP @50% IOU of 2% when using the best performing movement augmentation process rather than the raw image data (with similar results for the Kakadu Fish dataset). This is further supported by observing results at the species class level, where ‘rarer’ species classes (those with fewer than 400 annotations) generally showed improved performance when using movement augmentation approaches, whereas well‐labelled classes tended not to benefit as much (Table 4 and Figures S6–S9 in the Supporting Information). Altering the predicted detections using the ByteTrack algorithm had the worst performance across the Tassie BRUV and Kakadu Fish datasets it was trialled on. One possible reason for this is that the final step in the tracking approach no longer optimises the predictions based on the training data, rather it alters the predictions based on a ‘pre‐trained’ model in an unsupervised fashion.

**TABLE 3:** Predictive performance on the test sets for each dataset and method. Note that ‘Raw’ is using the original unmodified image data. The results of the best performing method for each dataset are bolded.

Data	Method	mAP @50% IOU	mAP @75% IOU	mAP @50:95% IOU
Deepfish (n=620)	Optical flow	**0.779**	0.544	0.530
	Background subtraction	0.751	0.472	0.469
	Frame differencing	0.764	**0.557**	**0.550**
	Raw	0.622	0.461	0.467
Wildcount (n=26,795)	Optical flow	0.969	0.938	0.884
	Background subtraction	0.970	0.938	0.889
	Frame differencing	0.970	0.940	0.887
	Raw	**0.977**	**0.952**	**0.904**
Kakadu Fish (n=8960)	Optical flow	0.937	0.833	0.684
	Background subtraction	0.939	0.820	0.686
	Frame differencing	0.933	0.817	0.679
	Tracking	0.906	0.697	0.594
	Raw	**0.940**	**0.835**	**0.690**
Tassie BRUV (n=1912)	Optical flow	0.727	0.512	0.471
	Background subtraction	0.729	0.520	0.491
	Frame differencing	**0.750**	**0.538**	**0.496**
	Tracking	0.642	0.378	0.388
	Raw	0.718	0.519	0.490

**TABLE 4:** PASCAL mAP @50% IOU (Padilla et al. 2021) results on the test split from the Tassy BRUV (TB), Wildcount (W), Deepfish (D) and Kakadu Fish (K) datasets for each predicted taxon, ranked based on the number of annotations, using raw annotated images (Raw), background subtraction (BS), optical flow (Flow), frame differencing (FD) and tracking (Track).

Data	Species class	Annotations	Raw	BS	Flow	FD	Track
K	Ambassis macleayi	9018	0.917	**0.924**	**0.922**	**0.918**	0.878
W	Eastern Grey Kangaroo	8404	0.982	0.974	0.971	0.968	NA
K	Neosilurus spp.	6062	0.940	**0.950**	**0.946**	**0.949**	0.881
TB	Scorpaeniformes	3843	0.844	0.827	**0.845**	0.838	0.792
K	*Amniataba percoides*	3066	0.971	0.970	0.968	0.956	0.948
W	Red Fox	3041	0.998	0.995	0.997	0.998	NA
W	Rabbit Hare	2455	0.995	**0.997**	0.993	0.993	NA
W	Brushtail Possum	2416	0.998	**1.000**	0.997	0.998	NA
W	Red‐necked Wallaby	2268	0.994	0.986	0.989	0.989	NA
W	Cat	2158	0.986	0.980	0.980	0.975	NA
W	Superb Lyrebird	1916	0.980	0.976	0.978	0.977	NA
W	Dog	1836	0.961	**0.970**	**0.971**	**0.980**	NA
K	Other	1811	0.942	0.930	0.934	0.931	0.928
W	Pig	1555	0.962	0.957	**0.962**	0.951	NA
W	Long‐nosed Bandicoot	1276	0.951	0.945	0.942	0.942	NA
W	Echidna	1096	1.000	0.992	1.000	1.000	NA
W	Brown Bandicoot	1075	0.947	0.922	0.934	**0.947**	NA
W	Koala	970	1.000	0.992	0.985	0.990	NA
W	Fallow Deer	907	0.992	**0.993**	0.990	**0.998**	NA
W	Euro	743	0.980	0.957	0.965	0.960	NA
W	Cervid Deer	603	0.985	0.961	0.968	0.960	NA
W	Rat	603	0.951	0.920	0.937	0.917	NA
W	Brush‐tailed Rock‐wallaby	554	0.981	0.953	0.971	**0.981**	NA
W	Common Wombat	486	1.000	1.000	0.989	0.989	NA
TB	Perciformes silver	452	0.777	0.738	0.698	0.709	0.551
W	Goat	431	0.976	0.904	0.976	0.952	NA
D	Fish	310	0.624	**0.750**	**0.782**	**0.767**	NA
W	Red‐legged Pademelon	303	0.945	**0.974**	**0.962**	**0.978**	NA
TB	Chyrosophyrs auratus	287	0.914	0.908	0.909	**0.939**	0.880
W	Red‐necked Pademelon	279	0.960	**0.999**	**0.979**	**0.972**	NA
TB	Carcharhiniformes	210	0.749	**0.798**	0.714	**0.906**	0.692
W	Horse	206	0.997	**1.000**	0.925	**1.000**	NA
W	Spotted‐tailed Quoll	129	0.998	**1.000**	**1.000**	0.958	NA
TB	Perciformes sandy	120	0.444	**0.511**	**0.568**	**0.618**	0.348
TB	Ray	115	0.575	**0.592**	**0.605**	0.570	0.515
TB	Moridae	102	0.744	**0.772**	0.731	**0.770**	0.700
TB	Tetradontiformes	93	0.703	**0.709**	**0.755**	0.678	0.677

*Note:* Prediction results that improved upon those using raw images alone are in bold. Although noisy, it seems that there is a trend between the ability for movement augmentation methods to improve prediction results and the number of annotations—or equivalently, the ‘rarity’ of the species (more bold results can be seen towards the bottom of the table).

The chosen tuning parameters differed considerably for each dataset (Table 2). The Kakadu Fish and Tassie BRUV datasets generally had large values for Δt and υ, as well as a direction based approach to frame differencing and PCA as the chosen augmentation method (Table 2). However, the converse was true for the Deepfish and Wildcount datasets, with small values for Δt and υ, an absolute value approach to frame differencing and replacing the red layer or all the colour layers being the generally preferred augmentation approach (Table 2). These differences could be explained by the fact that the Kakadu Fish and Tassie BRUV datasets had surrounding video data instead of surrounding images, with much smaller time increments between each image/frame. When applying background subtraction, a simple frame differencing based approach performed better than KNN across all four datasets.

## 4 Discussion

By comparing a large range of methods that are accessible and practical for ecologists to use on over 35,000 images and frames from a diverse range of terrestrial, marine and freshwater environments, we are able to guide ecologists on the usefulness of leveraging movement information to improve the detection accuracy of machine learning algorithms on environmental monitoring videos and camera traps.

Our results suggest that if a study has enough training data (>400 annotations per class at a minimum), an object detection algorithm might not be improved by augmenting the training data with movement information, as we found negligible difference in performance in these cases. Although we re‐trained an object detection algorithm to detect movement augmented images, this algorithm was still designed to maximise performance on large scale image datasets which do not contain movement augmented images. As such, the architecture of the algorithm (e.g., the number of hidden layers or the number of neurons per layer, etc.) may not be optimal for predicting movement, which may explain our findings. We also found little difference in performance between our movement augmentation approaches, even though they varied considerably in their complexity—in fact the simplest approach, frame differencing, arguably performed marginally better than the other augmentation approaches. Other researchers have found that combining background subtraction and/or optical flow with an object detection algorithm boosted predictive performance on the Fish4Knowledge Complex Scenes and LifeCLEF 2015 dataset (Salman et al. 2020; Jalal et al. 2020; Duhayyim et al. 2021). These datasets are however generated from very low resolution videos, which is no longer representative of the video technology of today (hence why they were not included in this study). Although movement augmentation methods performed well on the smaller sized datasets, researchers need to also consider the added time and computational resources needed to implement these methods in practice (noting that any video/images you want to predict using this method would also have to be augmented in the same way). In most cases, it is likely more beneficial to simply label additional training data as a means to improve predictive performance, rather than attempting to use alternative methods that incorporate movement information. However, these methods could be useful in situations where collecting more training data is exceedingly difficult (due to rare species, difficult access environments, resourcing, time constraints, etc).

It is tempting when applying a machine learning technique to use the same settings that were found to be optimal in a previous study. However, across our four datasets, we found that quite different strategies were optimal when choosing tuning parameters (Table 2) or when deciding on a strategy for incorporating movement information (Table 3). Thus, we recommend ecologists separately tune their own dataset rather than relying on previous experience.

We would not recommend combining a tracking algorithm with an object detection algorithm for the purpose of boosting detection scores on ecological monitoring data—on the two datasets where we were able to implement this approach, it performed more poorly than all other methods. The intended purpose of tracking algorithms is to connect neighbouring detections to track movement, rather than to improve the detection rate, and it would seem that these algorithms are best left to their intended purpose.

It is important to clarify that whilst this study was larger in scale than previous work investigating movement algorithms, we did ultimately analyse only four distinct datasets. Although many researchers have made their libraries of images and videos publicly available, these are typically not labelled in a way such that movement information can be utilised. In order to better generalise these results, it would be beneficial to apply these methods on a wider range of terrestrial and marine datasets. In particular, it would be interesting to understand whether the results we observed vary with environmental differences, species behaviour or even annotation quality. Although many methods were trialled that incorporate movement into prediction, this is far from an exhaustive list. There are also many more tuning parameters that could have been tweaked which may have improved the results beyond what we observed. For example, only one optical flow algorithm and one tracking algorithm were trialled, when there are a rapidly growing number of methods in the literature. It would also be interesting to apply more advanced methods, like a two‐stage detector that first leverages tracking algorithms to convert single‐frame training annotations into multi‐frame tubelets, and secondly feeds these tubelets into a ‘tubelet detector’. This would use richer movement information over longer periods of time; however, the additional step of creating tubelets could induce errors in the training data that could bias optimisation of the tubelet detector.

Finally, using object detection algorithms to automatically detect taxa in monitoring videos produces large volumes of new data on species occurrence, with multiple taxa typically detected with varying confidence scores across tens of thousands of frames in a video. This presents an exciting new opportunity—how to best use this richer data source to characterise fish communities? We look forward to seeing work in this direction in the future.

## Author Contributions

**Ben Maslen:** conceptualization (lead), formal analysis (lead), investigation (lead), methodology (lead), software (lead), validation (lead), visualization (lead), writing – original draft (lead). **Gordana Popovic:** conceptualization (equal), formal analysis (equal), investigation (equal), methodology (equal), supervision (equal), writing – review and editing (equal). **Dadong Wang:** conceptualization (equal), formal analysis (equal), investigation (equal), methodology (equal), resources (lead), supervision (equal), writing – review and editing (equal). **Andrew Jansen:** data curation (lead), resources (equal), writing – review and editing (equal). **David Warton:** conceptualization (equal), investigation (equal), methodology (equal), supervision (lead), writing – review and editing (equal).

## Conflicts of Interest

The authors declare no conflicts of interest.

## Supporting information


**Data S1:** ece371996‐sup‐0001‐supinfo.pdf.

## Data Availability

Four datasets were used for this study. The Kakadu Fish, Deepfish and Wildcount datasets are already publicly available (see Data preparation section in the Supporting Information for further details). The Tassie BRUV dataset that was generated for this publication is available as a benchmark dataset and can be accessed from the Dryad public repository https://doi.org/10.5061/dryad.sbcc2frf7. Code used to generate the results in this article is also available from the Github repository, https://github.com/BenMaslen/MCD.
