# Supplementary material for: The Motion Picture: Leveraging Movement to Enhance AI Object Detection in Ecology
Source: Ecol Evol. 2025 Aug 19;15(8):e71996. doi: 10.1002/ece3.71996 (PMC12364559; doi:10.1002/ece3.71996)
Supplement: Supplementary file 1 — Data S1: ece371996‐sup‐0001‐supinfo.pdf. [file ECE3-15-e71996-s001.pdf]

Supporting information

Supporting results

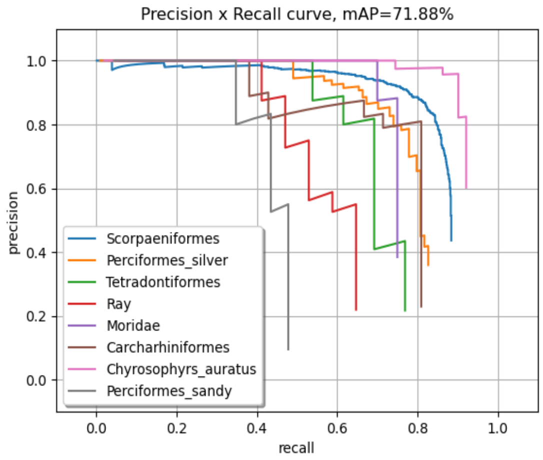

(a) RAW

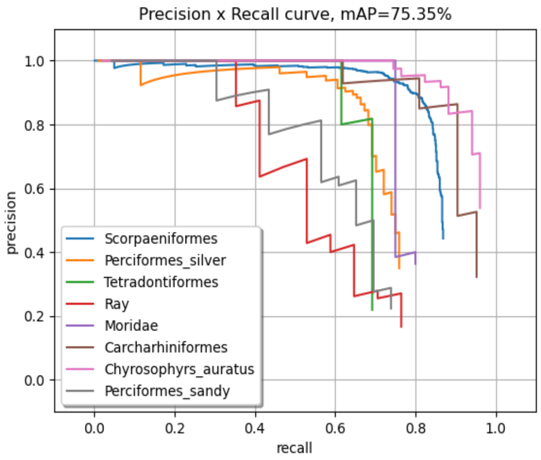

(b) Frame differencing

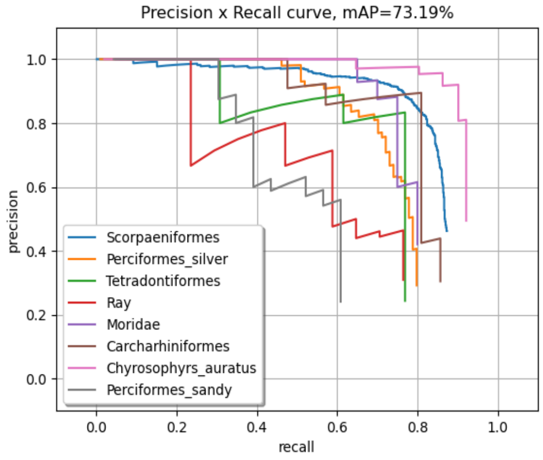

(c) Background subtraction

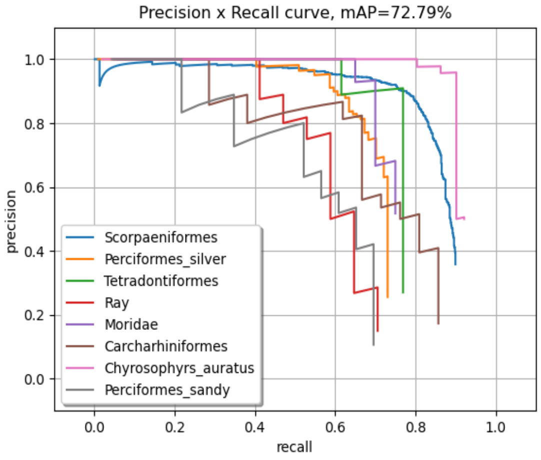

(d) Optical flow

Figure 6: Precision recall curves on the hold out test split from the Tassy BRUV dataset using the original raw annotated images (a), frame differencing (b), background subtraction (c), and optical flow (d). Note that overall mAP values differ slightly to those in Table 3, as PASCAL mAP was used to generate these curves (see Padilla et al., 2021, for further details).

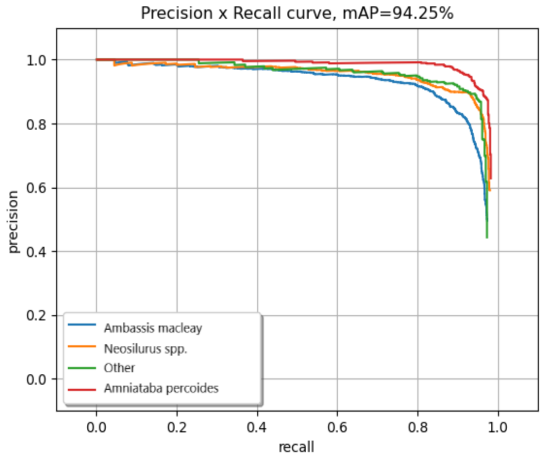

(a) RAW

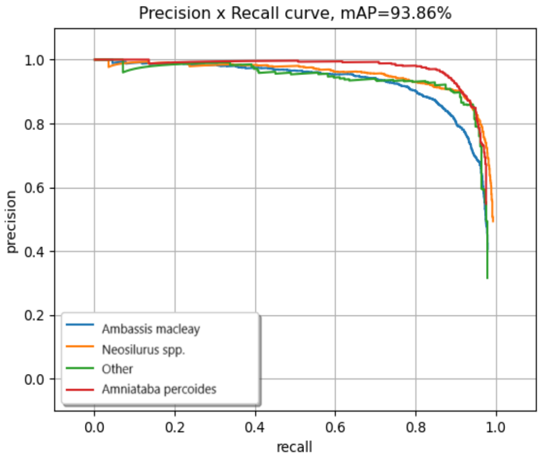

(b) Frame differencing

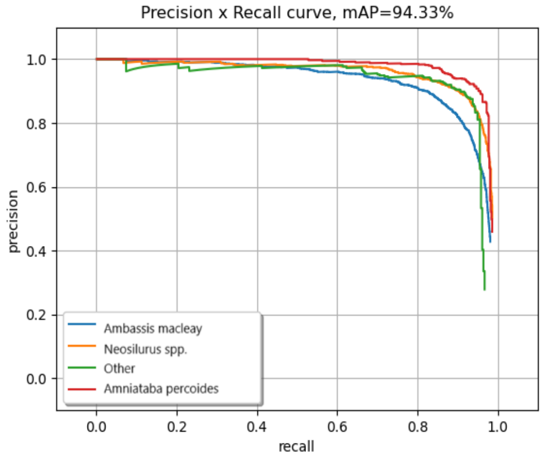

(c) Background subtraction

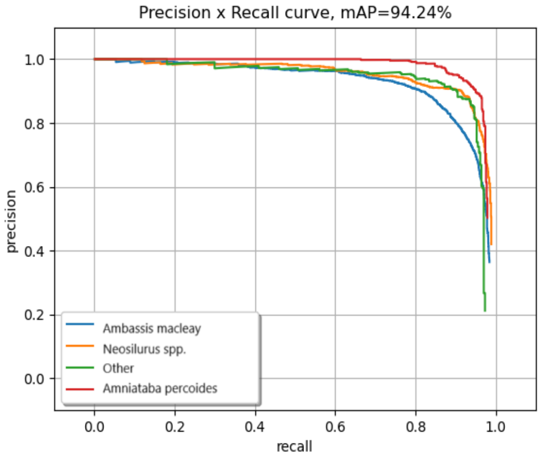

(d) Optical flow

Figure 7: Precision recall curves on the hold out test split from the Kakadu fish dataset using the original raw annotated images (a), frame differencing (b), background subtraction (c), and optical flow (d). Note that overall mAP values differ slightly to those in Table 3, as PASCAL mAP was used to generate these curves (see Padilla et al., 2021, for further details).

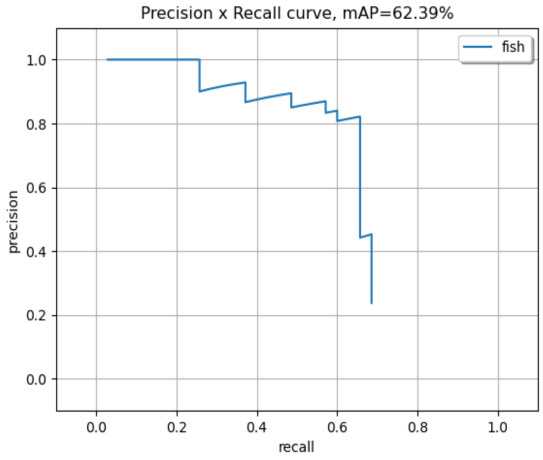

(a) RAW

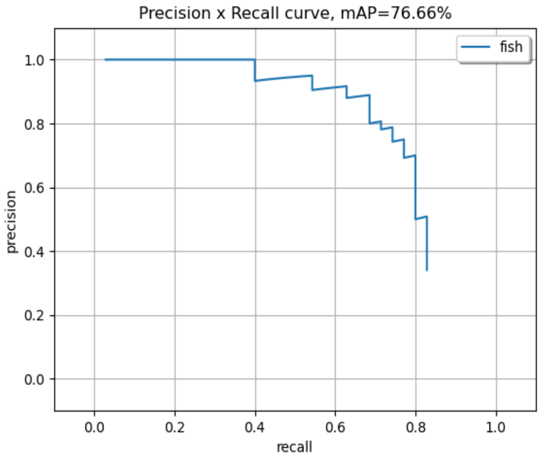

(b) Frame differencing

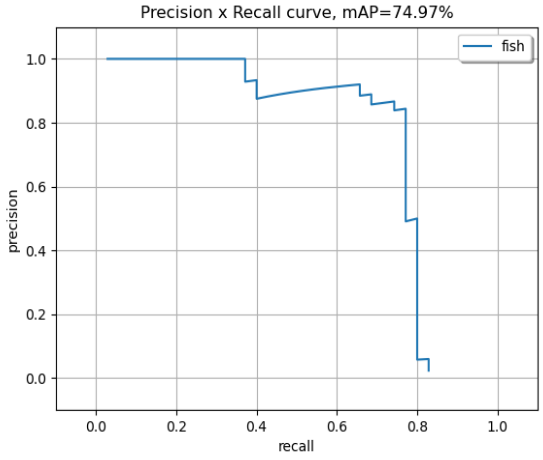

(c) Background subtraction

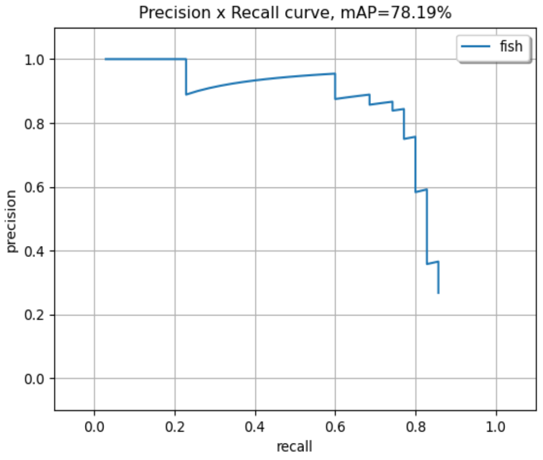

(d) Optical flow

Figure 8: Precision recall curves on the hold out test split from the Deepfish dataset using the original raw annotated images (a), frame differencing (b), background subtraction (c), and optical flow (d). Note that overall mAP values differ slightly to those in Table 3, as PASCAL mAP was used to generate these curves (see Padilla et al., 2021, for further details).

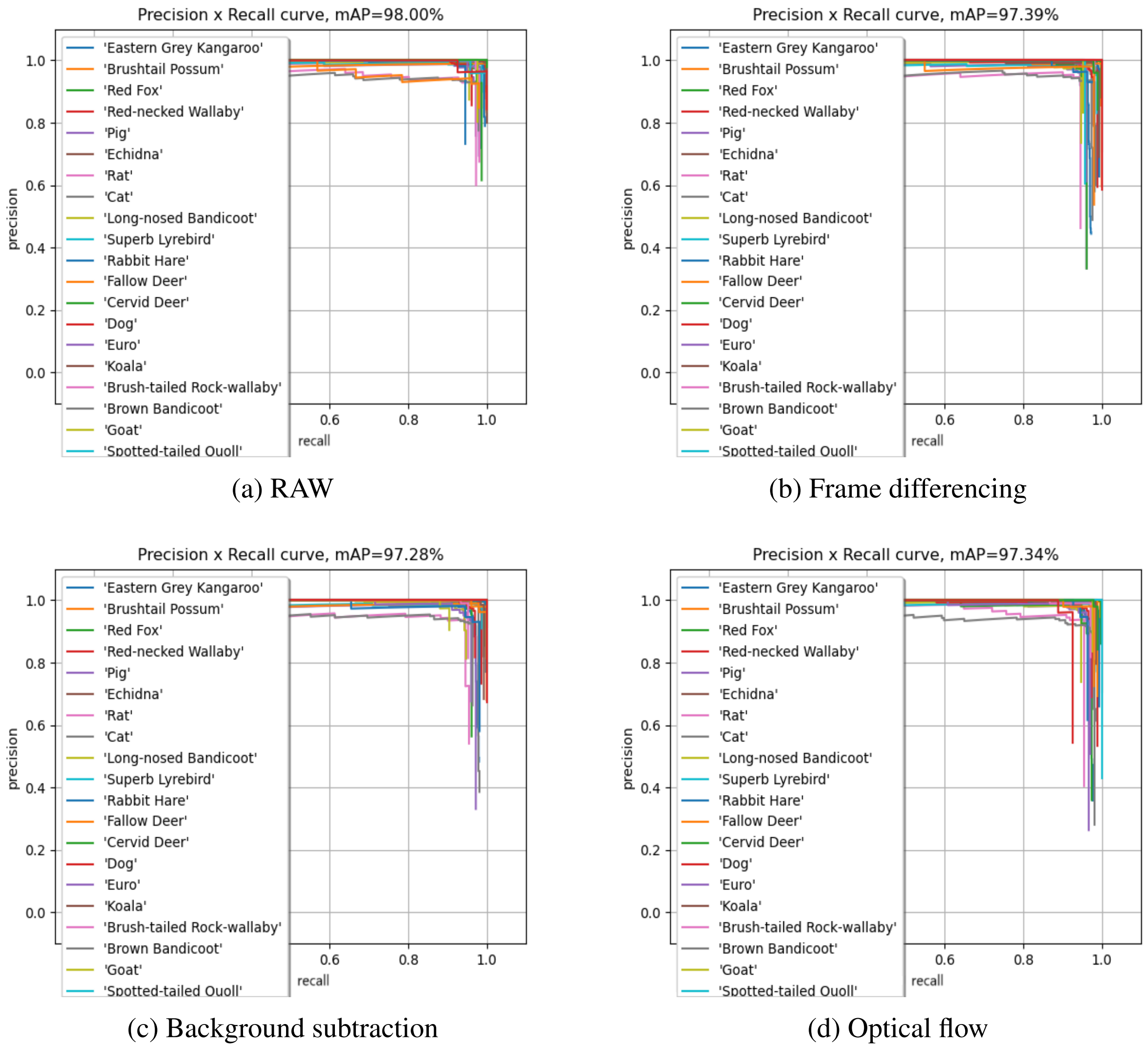

(a) RAW

(b) Frame differencing

(c) Background subtraction

(d) Optical flow

Figure 9: Precision recall curves on the hold out test split from the Wildcount dataset using the original raw annotated images (a), frame differencing (b), background subtraction (c), and optical flow (d). Note that overall mAP values differ slightly to those in Table 3, as PASCAL mAP was used to generate these curves (see Padilla et al., 2021, for further details).

Data preparation

Tassie BRUV

The Tassie BRUV dataset is an image dataset that we generated as a benchmark dataset using Baited Remote Underwater Videos (BRUVs) from chain moorings and environmentally friendly moorings in Tasmania. These videos were collected by CSIRO and OzFish volunteers as part of a separate study to observe if there are differences in the community abundance of fish species between chain and environmentally friendly moorings in this area (not yet published). This dataset can be accessed from the Dryad public repository https://doi.org/10.5061/dryad.sbcc2frf7.

We used 28 BRUVs from this dataset that were placed next to chain moorings (16), control locations (6) (locations within a site with no moorings), and environmentally friendly moorings (6). These BRUVs were located at either North West Bay (7), Battery Point (4) or Sandy Bay (17) in Tasmania. The start and ends of these videos were cropped such that the camera being lowered and raised from the sea floor was removed and a time stamp in the top left hand corner of the video was blacked out (Figure 1) to ensure it doesn't confound the effect of our measurements of movement. Then 50 frames were randomly selected from each of these videos and bounding boxes with fish id's to species level were labelled for each of them. Fish were detected using the videos as well as the frame image to help observe any camouflaged fish that were present. Then a further 100 frames were randomly selected from each of these videos and they were inspected if they contained any fish whose counts in the previous labelling process were less then 100 (referred to as 'rare fish'). Of the 2,800 frames, 512 had rare fish in them which were additionally labelled to boost the labels of rarer species in the dataset. This brought the total number of frames annotated to 1912, which were split into training (1340), test (380) and validation (192) sets. Unfortunately there were still many species with low counts, so multiple species were grouped into higher order taxonomic levels with similar morphological attributes (Supporting information Table 5). In total 5,222 fish annotations were generated, with 3,834 of these annotations being Platycephalus bassensis (Southern Sand Flathead), a benthic fish that lies on the sandy ocean floor, camouflaging itself by burying in the sandy sediment to ambush prey. There was also a clear baiting effect present in the videos with majority of fish being annotated close to the bait in front of the camera (Supporting information Figure 10).

Table 5: Species and Order taxonomic classification as well as the annotation grouping and total number of annotations for each fish species found present in the annotated frames. Moridae, Gobiidae, Pseudocaranx, Rajidae, Trygonoptera and Tetraodontidae fish could not be classified to species level and so are classified to genus or family level in the species column. Species in the orders Scorpaeniformes, Tetraodontiformes or Carcharhiniformes were grouped into these higher order taxonomic units, whilst 'Rays' were all grouped together being a part of the super order Batoidea. Perciformes were then grouped into three groups; Chyrosophyrs auratus (Australasian Snapper), Perciformes_sandy (benthic perciformes species with sandy colouration - Sillaginodes punctata & Gobiidae) and Perciformes_silver (pelagic perciformes species with silver colouration - Arripis truttaceus & Pseudocaranx)

Species	Order	Annotation grouping	Annotation counts
Cephaloscyllium laticeps	Carcharhiniformes	Carcharhiniformes	209
Mustelus antarcticus	Carcharhiniformes	Carcharhiniformes	1
Chyrosophyrs auratus	Perciformes	Chyrosophyrs auratus	287
Moridae	Gadiformes	Moridae	102
Gobiidae	Perciformes	Perciformes_sandy	4
Sillaginodes punctata	Perciformes	Perciformes_sandy	116
Arripis truttaceus	Perciformes	Perciformes_silver	417
Pseudocaranx	Perciformes	Perciformes_silver	35
Dasyatis brevicaudata	Myliobatiformes	Ray	3
Myliobatis australis	Myliobatiformes	Ray	19
Rajidae	Rajiformes	Ray	35
Spiniraja whitleyi	Rajiformes	Ray	56
Trygonoptera	Myliobatiformes	Ray	2
Helicolenus percoides	Scorpaeniformes	Scorpaeniformes	9
Platycephalus bassensis	Scorpaeniformes	Scorpaeniformes	3834
Brachaluteres jacksonianus	Tetraodontiformes	Tetradontiformes	6
Dicotylichthys punctulatus	Tetraodontiformes	Tetradontiformes	3
Meuschenia australis	Tetraodontiformes	Tetradontiformes	79
Tetraodontidae	Tetraodontiformes	Tetradontiformes	5

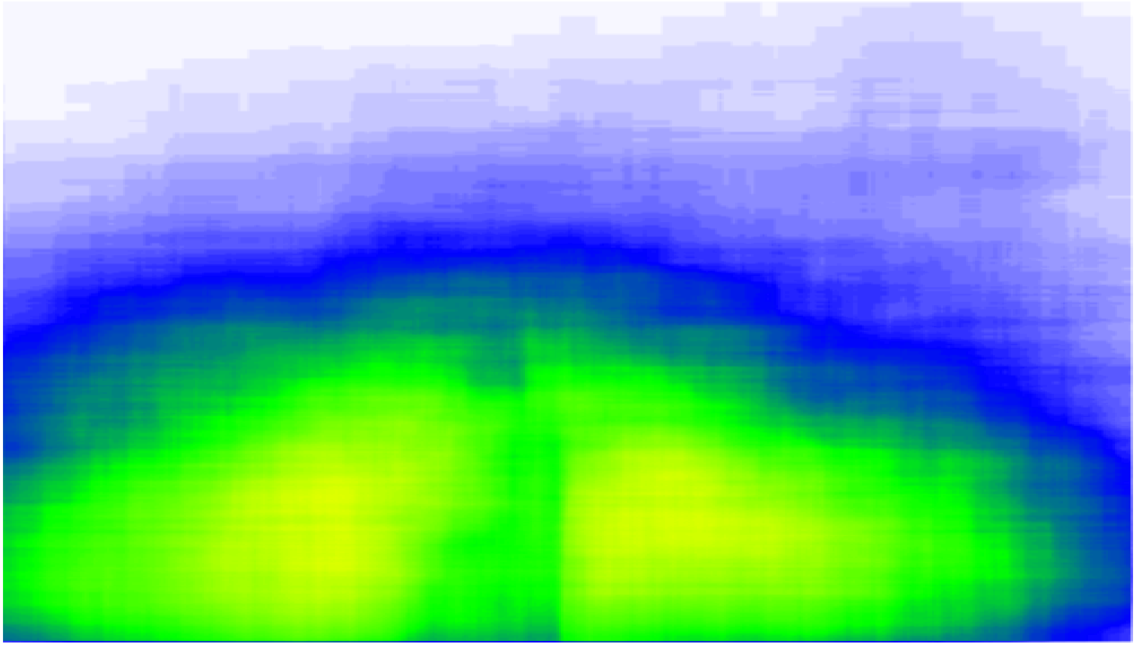

Figure 10: Annotation heatmap, depicting all 5,222 bounding box annotations of fish in the Tassie BRUV dataset.

Deepfish

The 'Deepfish' image dataset is a benchmark dataset with $40,000$ frames from underwater unbaited videos across 20 different habitats structures in tropical Australian waters (Saleh et al., 2020). Unfortunately, majority of the data is labelled to a fish/no fish level with only 620 frames being labelled with masks around the individual fish (310 of which are also 'empty' frames containing no fish). These frames come from 57 different frame sequences of varying lengths across 19 different habitats. The original test, training and validation split for the competing benchmark dataset had frames from the same sequence across multiple splits. The large dependency of images from the same sequence does not allow the test and validation sets to be true 'hold out' sets, allowing object detection algorithms trained on the training set to perfectly detect images in the test set. As such, we implemented a different split, where each image sequence was split into either a test (64), validation (68) or training (488) split, thus no images in one set was a part of an image sequence present in another set. In order to make the results more comparable to our other datasets, image masks were also converted to bounding boxes for detection.

Wildcount

The Wildcount dataset is an annotated camera trap dataset from two large scale and long term monitoring projects from the NSW National Parks and Wildlife Services (NPWS), the 'Wildcount' and 'Vertebrate Pest

Monitoring' projects along eastern NSW, Australia (McSorley et al., 2023; McHugh et al., 2022; NPWS, 2025). In total, 42,085 annotated images with bounding boxes and species id labels were generated, with 25,693 coming from the Wildcount project and 16,392 from the Vertebrate Pest Monitoring project. Camera traps from both of these projects are motion triggered, taking burst shots with multiple photographs in short succession. As such, movement can be obtained from this dataset by comparing a reference image to neighbouring images in a burst sequence (similar to comparing neighbouring frames in the monitoring video datasets). Of the original 42,085 images, 6,395 were not included as we did not have access to their surrounding images and another 8,895 were removed due to having large changes in brightness across neighbouring images in the burst camera sequence. This left a dataset of 26,795 images each with an annotated bounding box and id label from one of 24 classes of terrestrial vertebrate species split into training (17,428), test (5,331) and validation (4,036) sets. Some of these classes are groups of species that were morphological inseparable on camera traps such as the Mountain and Common Brush-Tailed Possums.

Kakadu Fish

The Kakadu Fish data consists of unbaited Remote Underwater Videos (RUVs) collected by the Office of the Supervising Scientist within the Australian Department of Climate Change, Energy, Environment and Water (SSD, 2011). The video's were collected during annual fish monitoring surveys between 2016 and 2018 at Mudginberri and Sandy Billabongs, Kakadu National Park, Northern Territory, Australia. Sampling occurred annually during the recessional flow period (late-wet-early-dry season) where the sampling design for each billabong consists of 5 transects with 10 RUVs per transect. Monitoring of fish communities is conducted to detect potential impacts from operational and rehabilitation operations at Ranger Uranium Mine.

From this fish monitoring project, freshwater ecologists labelled 44,112 frames, containing a total of 82,904 bounding box annotations of 23 species of freshwater fish (Jansen et al., 2024). However, the metadata relating which video and frame number each annotated frame was derived from was not available. This information (frame number and video name) is required to obtain measures of movement (e.g. when using neighbouring frames for frame differencing, we need to know where the neighbouring frames are). Therefore, we undertook a computationally intensive matching process to attempt to match the 44,112 annotated frames to a video frame in one of the 352 RUVs available. Each RUV was approximately 77 minutes in duration and 50 frames per second, thus having $\sim$231,000 frames per video. A portion of the videos were originally collected upside down, whilst the annotated frames that were sourced from these videos were corrected to be right way up. As such, the matching process was run twice to account for upside down videos.

Each frame in the annotated dataset consisted of of $1080 \times 1920 \times 3 = 6,220,800$ pixels, and the process of extracting and labelling each frame altered the pixel data slightly (in a somewhat random fashion), meaning that perfect matches could not be found and we had to instead look for the best 'imperfect' match. Root Mean Square Error (RMSE) was used as a measure to compare the pixel values from the annotated and video frames. Thus, our approach searched for the best imperfect match for each set of 6,220,800 pixels from 44,112 annotated frames to $\sim$162 million video frames, resulting in a total search of of $\sim 7 \times 10^{12}$ image matches or $\sim 4 \times 10^{19}$ pixel matches.

This task was highly computationally intensive, particularly to load large amounts of image and video data for analysis. To achieve this high workload, data was analysed on the CSIRO HPC Petrichor and split into 2 stages. The first stage was to compare each annotated frame to a set of frames extracted every 5 minutes from each of the 1707 RUVs, resulting in the most likely videos that each annotated image was derived from. The second stage was to compare each image to every frame in the top three videos that were matched in stage 1. Although not perfect, a total of 8,960 images were able to be matched to a frame in a video. We also did not have access to all the surrounding videos where annotated frames were sourced from. As such, annotated frames may not have been able to be matched due to missing video files, not finding the right video in stage 1 of the matching algorithm or due to image alterations in the labelling and extracting of the original annotated frames.

The 8,960 annotated frames were then split into training (5,840), test (1,800) and validation (1,320) splits. These remaining frames had 19,957 annotations from 19 species, where species with total annotation counts less then 500 were grouped into 'other' (Supporting information Table 6), leading to a total of 4 classes.

Table 6: Species, annotation grouping and annotation counts for the Kakadu Fish dataset. Any species whose annotation counts were less than 500 were grouped together in the 'Other' category. There are a total of 19,957 annotation with 1,811 annotations from the 'Other' category.

Species	Annotation grouping	Annotation count
Ambassis macleayi	Ambassis macleayi	9,018
Neosilurus spp.	Neosilurus spp.	6,062
Amniataba percoides	Amniataba percoides	3,066
Glossamia aprion	Other	479
Sycomistes butleri	Other	354
Toxotes chatareus	Other	315
Strongylura krefftii	Other	196
Lates calcarifer	Other	157
Neoarius spp.	Other	98
Nematalosa erebi	Other	87
Scleropages jardinii	Other	49
Leiopotherapon unicolor	Other	20
Hephaestus fuliginosus	Other	17
Liza ordensis	Other	10
Ambassis agrammus	Other	9
Megalops cyprinoides	Other	8
Oxyeleotris lineolata	Other	5
Craterocephalus sturcusmuscarum	Other	4
Melanotaenia splendida inornata	Other	3
